# Pharmaceutical Applications of 3D Printing

**DOI:** 10.3390/pharmaceutics18091059

**Published:** 2026-08-26

**Authors:** Philippe Espeau

**Affiliations:** CNRS, INSERM, Chemical and Biological Technologies for Health Group (UTCBS), Université Paris Cité, 75006 Paris, France; philippe.espeau@u-paris.fr

**The context of 3D printing in the pharmaceutical sector and its significance**

The history of three-dimensional (3D) printing dates back to the 1980s with the emergence of the first additive manufacturing technologies, including Selective Laser Sintering (SLS) and Fused Deposition Modelling (FDM) [1,2,3]. In 1989, Scott Crump, co-founder of Stratasys, invented and patented FDM technology, which has since become one of the most widely adopted additive manufacturing techniques [4].

The rapid evolution of 3D printing has created unprecedented opportunities for the development of innovative medical devices and personalized drug delivery systems. Unlike conventional pharmaceutical manufacturing, which relies on large-scale production and standardized dosage forms, additive manufacturing enables the on-demand fabrication of patient-specific medicines in a flexible, cost-effective, and decentralized manner [5]. By depositing materials layer by layer according to a digital three-dimensional model, 3D printing allows the production of dosage forms with customized geometries, drug loads, and spatial distributions of active pharmaceutical ingredients (APIs). This versatility enables the design of formulations with tailored drug-release profiles adapted to individual therapeutic requirements [6].

The ability to personalize medicines is particularly valuable in pediatric pharmacotherapy, where commercially available strengths are often unsuitable. Three-dimensional printing also facilitates the incorporation of multiple APIs into a single dosage form, simplifying complex treatment regimens, improving patient adherence, and supporting the transition toward precision medicine [7,8].

A landmark in pharmaceutical additive manufacturing was reached in August 2015 with the approval by the U.S. Food and Drug Administration (FDA) of Spritam^®^ (levetiracetam), developed by Aprecia Pharmaceuticals using ZipDose^®^ technology. As the first FDA-approved 3D-printed medicine, Spritam^®^ demonstrated the clinical feasibility of additive manufacturing by producing highly porous tablets that rapidly disintegrate with a small volume of water, thereby facilitating administration, particularly in pediatric and geriatric populations [9].

Despite these remarkable advances, several scientific, technological, and regulatory challenges continue to hinder the widespread implementation of pharmaceutical 3D printing. Further optimization is required regarding pharmaceutical-grade printable materials, printing hardware and software, process robustness, and the mechanical properties of printed dosage forms [10]. Industrial adoption will also depend on achieving consistent dose accuracy, batch-to-batch reproducibility, multi-material printing capability, increased production throughput, and full compliance with Good Manufacturing Practice (GMP). In parallel, regulatory frameworks must continue to evolve to ensure the quality, safety, and efficacy of personalized medicines produced through additive manufacturing.

Nevertheless, 3D printing is expected to complement and in selected applications potentially replace conventional manufacturing methods, particularly for personalized therapies, pediatric medicines, orphan drugs and small-batch production. By enabling the manufacture of individualized dosage forms at the point of care or in specialized facilities, additive manufacturing represents a promising strategy for advancing personalized pharmacotherapy and improving patient outcomes [4].

Pharmaceutical 3D-printing technologies can generally be classified into four major categories: inkjet-based systems, nozzle deposition systems, electromagnetic systems and ultrasonic systems. Each category comprises several printing techniques that differ in their operating principles, printable materials and pharmaceutical applications [11,12].

**An overview of the articles published in this Special Issue**

This Special Issue, Pharmaceutical Applications of 3D Printing, provides a comprehensive overview of recent developments illustrating the expanding role of additive manufacturing in pharmaceutical sciences. It comprises three review articles and eight original research papers that collectively demonstrate the versatility of 3D printing for personalized medicine and advanced biomedical applications.

The three review articles highlight complementary aspects of pharmaceutical 3D printing. The first review, by Samir I. Paipa-Jabre-Cantu et al. discusses the role of 3D printing in pharmaceutical manufacturing, with particular emphasis on personalized oral drug-delivery systems for the treatment of central nervous system disorders [13]. The remaining two reviews focus on pediatric applications from distinct but complementary perspectives. Krisztina Petrinca et al. examines the regulatory challenges associated with the development and implementation of 3D-printed medicines for children [14], whereas Veronica Ianno et al. reviews the technological advantages of Fused Filament Fabrication (FFF) for manufacturing personalized pediatric dosage forms [15]. Together, these reviews provide a balanced overview of both the regulatory and technological issues that will shape the future of personalized pediatric medicines.

The eight original research papers further illustrate the breadth of pharmaceutical and biomedical applications of additive manufacturing. Alexandrina Druta et al. report the development of a porous palladium-functionalized silica catalyst produced by 3D printing for the synthesis of novel isatin derivatives with potential anticancer activity [16]. The increased porosity achieved through additive manufacturing enhances catalytic performance compared with conventionally manufactured structures.

Wouter Pannekoek et al. investigate medication handling practices in community pharmacies, nursing homes and a pediatric hospital [17]. The study reveals that a considerable proportion of medicines require manipulation before administration, emphasizing the potential value of 3D printing for producing individualized dosage forms better suited to patient needs.

Piyush Garg et al. present the development of 3D-printed contact lenses capable of sustained poly(vinyl alcohol) release for the treatment of dry eye disease, significantly extending therapeutic duration compared with conventional eye drops [18].

Farzana Khan Rony et al. compare Fused Deposition Modelling (FDM) with pressure-assisted micro syringe extrusion for manufacturing fenofibrate-loaded oral films [19]. Both techniques successfully produce formulations with high drug loading and markedly improved dissolution profiles.

Carlos Tamarit-Martínez et al. investigate antibiotic-loaded polylactic acid (PLA) implants fabricated by FDM for the prevention and treatment of prosthetic joint infections [20]. The authors demonstrate that print layer thickness is a key parameter influencing antibiotic release kinetics.

Martin Cseh et al. employ stereolithography to fabricate dissolvable microneedles containing dexamethasone [21]. The proposed manufacturing approach improves device stability while substantially reducing production time, representing an attractive strategy for personalized drug delivery.

Young-Jin Kim et al. evaluate the influence of mini-tablet geometry on the release kinetics of theophylline following FDM printing [22]. The results indicate that dose adjustment can be achieved by modifying tablet size and shape while maintaining controlled drug release, highlighting the flexibility of additive manufacturing for individualized therapies.

Finally, Jonas Lenhart et al. explore direct powder extrusion for the manufacture of loperamide tablets [23]. By adjusting drug loading and incorporating sorbitol as a plasticizer, the authors successfully modulate drug-release profiles while obtaining homogeneous amorphous solid dispersions, demonstrating the potential of this emerging technology for customized pharmaceutical manufacturing.
